# Serum vitamin D concentrations and sleep disorders: insights from NHANES 2011–2016 and Mendelian Randomization analysis

**DOI:** 10.1007/s11325-024-03031-2

**Published:** 2024-05-13

**Authors:** Junjie Jiang, Hanyu Tan, Zhongfang Xia, Jun Li, Shuang Zhou, Tao Huang

**Affiliations:** Department of Otolaryngology, Wuhan Children’s Hospital, Tongji Medical College, Huazhong University of Science & Technology, Wuhan, 430016 Hubei Province People’s Republic of China

**Keywords:** Sleep disorders, Serum vitamin D, National Health and Nutrition Examination Survey (NHANES), Mendelian Randomization (MR)

## Abstract

**Objective:**

This investigation seeks to examine the association between serum vitamin D concentrations and the prevalence of sleep disorders, additionally elucidating the causal relationship via Mendelian Randomization (MR) analysis.

**Materials and methods:**

This research employed data from the National Health and Nutrition Examination Survey (NHANES) 2011–2016, focusing on adults aged 20–50 years reporting sleep disorders. The research encompassed 4913 American adults. Weighted multivariable logistic regression models and cubic spline analyses were utilized to evaluate the association between serum vitamin D concentrations and the incidence of sleep disorders. Additionally, a two-sample Mendelian Randomization analysis was performed to evaluate the potential causal link between serum vitamin D concentrations and the risk of sleep disorders.

**Results:**

Within the 2011–2016 NHANES cohort of the U.S. population, a notable inverse association was detected between serum vitamin D concentrations and sleep disorders (*β* =  − 3.81, 95% CI: − 6.10 to − 1.52, *p* = 0.003). After multivariate adjustments, a higher incidence of sleep disorders was associated with lower vitamin D Concentrations (OR 1.52, 95% CI 1.10–2.10, trend *p* = 0.014). Restricted cubic spline regression analysis indicated a linear association between serum vitamin D concentrations and sleep disorders(non-linearity *p* > 0.05). Lastly, the two-sample MR analysis yielded evidence supporting a potential causal connection between serum vitamin D concentrations and sleep disorders, with each unit increase in genetically predicted serum vitamin D reducing the odds ratio to 0.78 (95% CI 0.61–0.99, *p* = 0.044).

**Conclusions:**

These results imply that lower vitamin D concentrations in the population might correlate with a heightened risk of sleep disorders, suggesting the importance of considering vitamin D supplementation when treating sleep disorders.

**Supplementary Information:**

The online version contains supplementary material available at 10.1007/s11325-024-03031-2.

## Introduction

Sleep is a fundamental and essential physiological activity. Insufficient sleep duration, or the presence of diseases that interfere with sleep quality, can lead to sleep disorders [1]. Globally, approximately one billion people suffer from sleep disorders, with the incidence of this condition continually rising, significantly impacting the quality of life for many [2]. Sleep disorders represent a major, under-recognized issue that leads to a variety of other health and societal problems. It has been demonstrated that sleep disorders are closely associated with a range of health concerns, including hypertension, type 2 diabetes, obesity, cardiovascular diseases, and an increased risk of overall mortality [3–6]. Simultaneously, sleep disorders also incur a substantial economic burden. Researchers in Australia have found that in the year 2004 alone, the economic cost of sleep disorders amounted to around 4.5 billion Australian dollars, representing approximately 0.8% of Australia’s Gross Domestic Product (GDP) [7].

Vitamin D is an important steroid hormone that plays a key role in regulating the levels of calcium and phosphorus in the body, which are extremely important for the development of bone tissue [8]. Its deficiency, a widespread health concern, has been linked to multiple acute and chronic health conditions, including sleep disorders [9–12]. Currently, several epidemiological studies have assessed the relationship between vitamin D and sleep disorders. Research indicates that vitamin D deficiency is a common occurrence among patients with sleep disorders, potentially serving as one of the causes of sleep disturbances; furthermore, the study by Majid et al. suggests that supplementation with vitamin D can enhance sleep quality and increase the duration of sleep for subjects suffering from sleep disturbances [13, 14]. However, some scholars have presented a differing view, arguing that there is no correlation between the two [15]. Presently, the relationship between serum vitamin D concentrations and sleep disorders remains inadequately explored and understood.

This study addresses this gap by investigating the potential correlation employing data from the National Health and Nutrition Examination Survey (NHANES) spanning 2011–2016. This study utilizes MR analysis, drawing on data from Genome-Wide Association Studies (GWAS) regarding serum vitamin D and sleep disorders, to determine the potential causal influence of serum vitamin D on the risk of sleep disorders.

## Materials and methods

### Data source

The investigation utilizes data from three successive iterations of the National Health and Nutrition Examination Survey (NHANES), encompassing the timeframes of 2011–2012, 2013–2014, and 2015–2016. The survey encompassed questionnaires, examination data, and laboratory test results and is conducted with the approval of the National Center for Health Statistics (NCHS) Institutional Review Board. Biennial data releases ensure up-to-date information, and participation is contingent on written informed consent from all respondents.

**Fig. 1:**
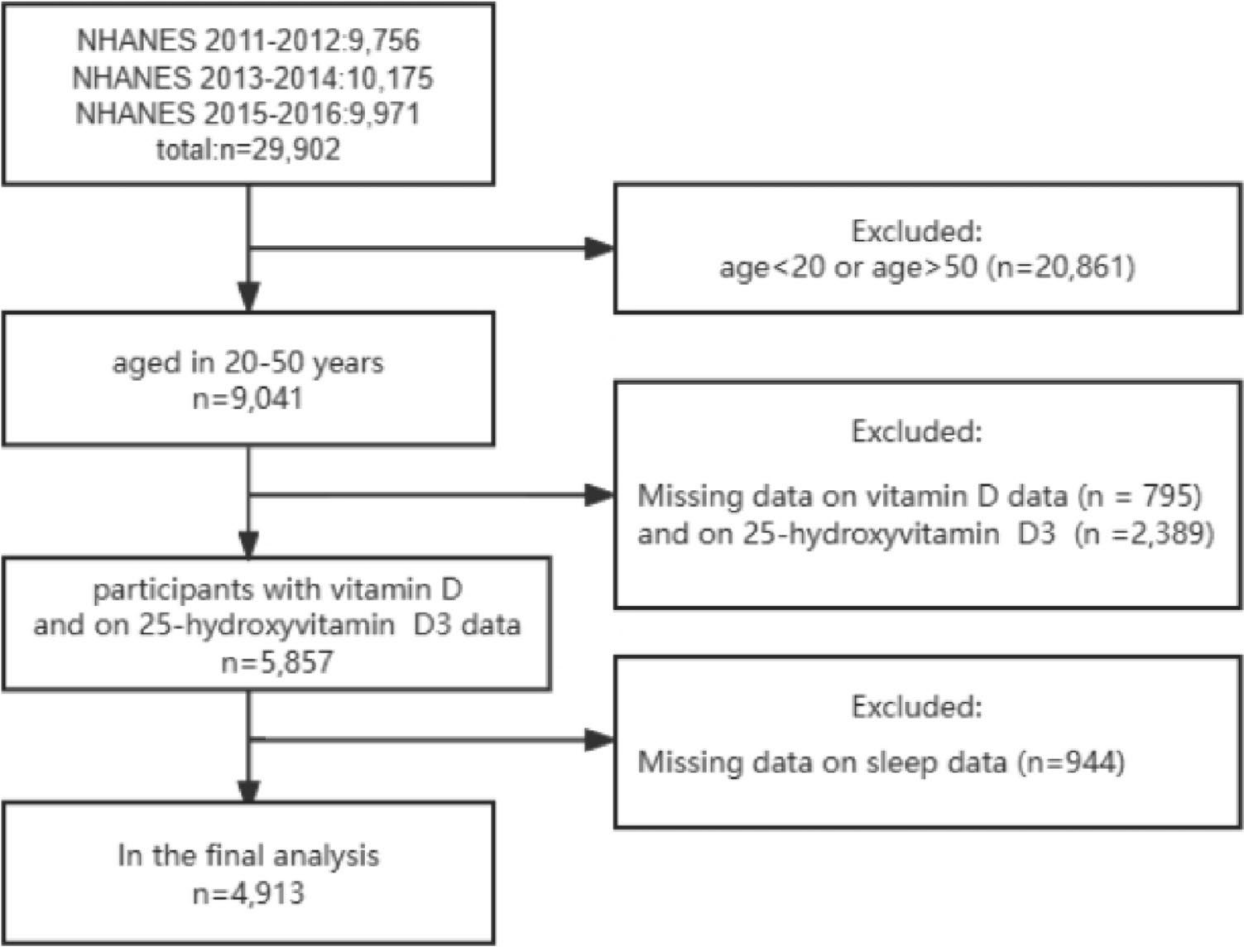
Flowchart of participants included in analyses

Between 2011 and 2016, the NHANES survey engaged 29,902 participants. Our analysis excluded individuals based on specific criteria: those not within the 20–50 age range (20,861 participants), absence of data on 25-hydroxyvitamin D3 (2,389 participants) and other related vitamin D metrics (795 participants), and incomplete information on sleep disorders (944 participants). This resulted in a final sample of 4913 for our study. Figure 1 depicts the selection process.

### Sleep disorders

Data on sleep disorders were gathered from participants’ responses to a specific query: “Over the last 2 weeks, how often have you been bothered by the following problems: trouble falling or staying asleep, or sleeping too much?” A sleep disorder was identified if the answer was “more than half the days” or “nearly every day.” Conversely, responses of “several days” or “not at all” indicated the absence of a sleep disorder.

### Laboratory measures

Ultra-performance liquid chromatography-tandem mass spectrometry (UHPLC-MS/MS) was employed for the quantification of 25-hydroxyvitamin D in human serum. The lower limit of detection (LLOD) for 25-hydroxyvitamin D3 was established at 2.23 nmol/L. The comprehensive methodology for this analytical procedure is extensively delineated in other scholarly publications. For values below the LLOD, the calculation employed was the square root of LLOD divided by two (LLOD/√2).

### Covariates

Continuous variables in the study encompassed age, body mass index (BMI), along with systolic and diastolic blood pressure. The study categorized variables as follows: Populations or ethnic groups are categorized into four distinct segments: Mexican Americans, non-Hispanic whites, non-Hispanic blacks, and other races. Gender included male and female. Educational attainment is categorized as either incomplete or complete with respect to upper secondary education. In categorizing marital status, the amalgamation of marriage and cohabitation into a single category is contrasted with the distinct classification of being single, divorced, widowed, or separated. Alcoholism, smoking, poverty-to-income ratio, high blood pressure, and diabetes are categorically classified as “yes” or “no.” Participants’ ages were classified into three categories: 20–29, 30–39, and 40–50. Obesity was defined as a BMI ≥ 30 according to Centers for Disease Control and Prevention (CDC) guidelines. Alcohol status was ascertained based on participants’ responses to the query, which inquired whether they had consumed 12 or more alcoholic drinks in the preceding year. Participants who responded affirmatively were classified as alcohol users, and conversely for those who answered negatively. Smoking status was assessed using the question: Do you now smoke cigarettes, with respondents answering “Every day” or “Some days” being considered as current smokers. The poverty-income ratio was delineated as ≥ 1.3 for non-poverty status and < 1.3 for poverty status. Each participant had three to four consecutive blood pressure levels taken. Systolic (SBP) and diastolic blood pressure (DBP) values were computed as the average of all extant readings. Hypertension was identified when SBP was ≥ 140 mmHg and DBP was ≥ 90 mmHg [16, 17].

## Mendelian Randomization (MR) analysis

### The concept of MR analysis

**Fig. 2:**
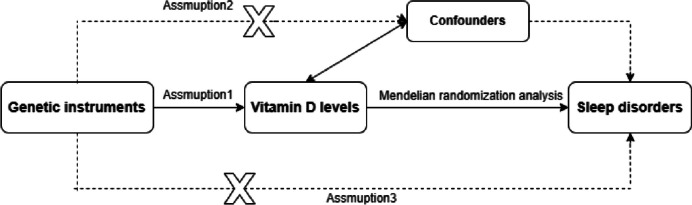
Mendelian Randomization flowchart. MR three major assumptions: (1) the need for genetic variation correlated with the level of 25(OH)D metabolite; (2) this correlation should remain uninfluenced by confounding variables; (3) such genetic variations should exert an impact on sleep disorders solely via the levels of the 25(OH)D metabolite

Given the random distribution of genetic variation during embryonic development and its independence from environmental influences. Mendelian Randomization (MR) analysis demonstrates a reduced susceptibility to reverse causality and confounding issues [18, 19]. Consequently, Mendelian Randomization (MR) analysis is utilized to ascertain single nucleotide polymorphisms (SNPs) linked to 25(OH)D metabolites and sleep disorders, to assess the risk and relationship between 25(OH)D metabolite levels and sleep disorders; 25(OH)D metabolites are serum vitamin D. The MR methodology is delineated in Fig. 2.

For the exposure dataset of serum vitamin D, we obtained GWAS analysis results involving 441,291 individuals of European descent to generate instrumental variables (IVs) (https://gwas.mrcieu.ac.uk/datasets/ieu-b-4812/). Concerning the GWAS outcome dataset, data pertaining to sleep disorders were procured from a separate GWAS analysis, encompassing 216,454 individuals of European ancestry, including 2628 cases and 213,826 controls (https://gwas.mrcieu.ac.uk/datasets/finn-b-F5_SLEEP/).

### Selection of SNPs for MR analysis

Initially, independent genetic variants exhibiting genome-wide significance (*p* < 5 × 10^−8^) were chosen from the pertinent datasets as potential instrumental variables correlated with 25-hydroxyvitamin D. Then, those SNPs with linkage disequilibrium (LD) coefficient < 0.001 and distances greater than 10,000 kb were selected as the LD reference group based on the European 1000 genomic dataset [20, 21]. We then screened for confounders by PhenoScanner, and BMI-related SNPs were excluded because BMI was associated with vitamin D concentrations and risk of sleep disturbances [22].

### MR analysis

In this Mendelian Randomization (MR) analysis, version 4.3.2 of R software and the TwoSampleMR package were utilized. The primary method used in the analysis was inverse variance weighted (IVW) [23].

## Statistical analysis

Continuous variables were reported as weighted means accompanied by standard errors, while categorical variables were presented in terms of frequencies and corresponding weighted percentages.

Initially, the baseline characteristics of the participants were delineated. Subsequently, weighted univariate and multivariate linear regression analyses were conducted to explore the relationship between vitamin D concentrations and various study variables, with the objective of identifying factors that influence vitamin D concentrations. Thirdly, the logistic regression model was employed to compute the adjusted odds ratios (OR) and 95% confidence intervals (CI) pertaining to the association between vitamin D concentrations and sleep disorders. Three covariate models were constructed as follows: Model 1 without adjustment for variables. In Model 2, adjustments were made for age, sex, and race/ethnicity as categorical variables. Model 3 was adjusted for all variables, while also assessing the trend across quartiles of vitamin D. To determine the linearity of the association between vitamin D concentrations and sleep disorders, restricted cubic spline regression was utilized. All statistical analyses, encompassing the ones delineated herein, were executed utilizing R software, version 4.3.2. For each analysis, a *p*-value of less than 0.05 was deemed to be statistically significant.

## Results

### Baseline characteristics of participants

The study encompassed 4,913 participants, with an average age of 34.94 ± 0.35 years; the largest age group was those aged 40–50, comprising 36.41%. A significant majority, about 86%, had at least a high school education. The gender distribution was nearly even, with males representing 50.42% and females 49.58%. The average serum vitamin D level among participants was 61.80 nmol/L (Table 1).

**Table 1:** Descriptions of study individuals’ characteristics

Variables	*N* = 4913
Age, (years) (%)	34.94 ± 0.35
20–29	1608 (33.25)
30–39	1578 (30.34)
40–50	1727 (36.41)
Race/ethnicity (%)	
Mexican American	663 (11.34)
Non-Hispanic white	1919 (61.14)
Non-Hispanic Black	1042 (11.56)
Other race	1289 (16.95)
Gender	
Male	2464 (50.42)
Female	2449 (49.58)
Married/live with partner	
Yes	2846 (60.61)
No	2067 (39.39)
Education level (%)	
Below high school	860 (14.17)
High School or above	4053 (85.83)
Poverty income ratio (%)	
Poor	1656 (25.53)
Not poor	3257 (74.47)
Body mass index (kg/m [2])	28.69 ± 0.18
Obesity	
Yes	1758 (35.34)
No	3155 (64.66)
Systolic blood pressure (mm Hg)	114.1 ± 0.64
Diastolic blood pressure (mm Hg)	69.384 ± 0.43
Smoking (%)	
Yes	1152 (22.42)
No	3761 (77.58)
Alcohol use (%)	
Yes	3752 (81.20)
No	1157 (18.80)
DM history	
Yes	361 (5.75)
No	4548 (94.25)
Hypertension history (%)	
Yes	1112 (21.69)
No	3801 (78.31)
25(OH)D (nmol/L)	61.807 ± 1.1925
Sleep disorder	
Yes No	690 (13.65) 4223 (86.35)

Continuous variables are presented as mean ± standard error (SE); categorical variables are presented as counts (weighted percentage). Poverty income ratio for not poor was defined as ≥ 1.3 and for poor was defined as < 1.3. Obesity was defined as when body mass index ≥ 30 kg/m^2^

*DM* diabetes mellitus, *25(OH)D* 25 hydroxy vitamin D3

### Associations between vitamin D and study variables

In the univariate linear regression analysis, significant correlations were observed between serum vitamin D concentrations and various factors including age, race, gender, marital status, education, poverty-income ratio, obesity, and alcohol consumption, as well as histories of diabetes, hypertension, and sleep disturbances. The adjusted multivariate analysis further revealed a significant inverse association between sleep disturbances (*β* =  − 3.61, 95% CI − 5.89, − 1.34) and serum vitamin D concentrations (*p* < 0.05), as detailed in Table 2.

**Table 2:** Linear regression analysis between vitamin D and study variables

	Univariate		Multivariate	
Variables	*β* (95% CI)	*P* value	*β* (95% CI)	*P* value
Age (years) (%)				
20–29	Ref		Ref	
30–39	0.27 (− 2.18, 2.72)	0.832	0.06 (− 2.18, 2.30)	0.960
40–50	4.77 (1.81, 7.73)	0.004	3.78 (1.12, 6.43)	0.012
Race/ethnicity (%)				
Mexican American	Ref		Ref	
Non-Hispanic white	19.45 (16.65, 22.25)	< 0.001	17.36 (14.63, 20.09)	< 0.001
Non-Hispanic Black	− 9.44 (− 12.76, − 6.13)	< 0.001	− 9.13 (− 12.42, − 5.83)	< 0.001
Other race	5.14 (1.92, 8.36)	0.004	3.74 (0.38, 7.10)	0.043
Gender				
Male	Ref		Ref	
Female	3.77 (2.09, 5.45)	< 0.001	4.86 (3.21, 6.51)	< 0.001
Married/live with partner				
Yes	Ref		Ref	
No	− 4.54 (− 6.88, − 2.20)	< 0.001	− 1.65 (− 3.88, 0.58)	0.165
Education level (%)				
Below high school	Ref		Ref	
High School or above	6.34 (3.15, 9.52)	< 0.001	0.23 (− 2.26, 2.73)	0.857
Poverty income ratio (%)				
Poor	Ref			
Not poor	7.76 (5.82, 9.70)	< 0.001	2.72 (0.46, 4.98)	0.030
Body mass index (kg/m^2^)	− 0.74 (− 0.88, − 0.59)	< 0.001	–	–
Obesity				
Yes	Ref			
No	8.98 (6.78, 11.17)	< 0.001	7.22 (5.33, 9.11)	< 0.001
Systolic blood pressure (mmHg)	− 0.18 (− 0.24, − 0.12)	< 0.001	–	–
Diastolic blood pressure (mmHg)	− 0.05 (− 0.16, 0.16)	0.386	–	–
Smoking (%)				
Yes	Ref		–	–
No	1.93 (− 0.79, 4.65)	0.173	–	–
Alcohol use (%)				
Yes	Ref		Ref	
No	− 6.36 (− 9.22, − 3.51)	< 0.001	− 2.78 (− 4.74, − 0.81)	0.013
DM history				
Yes	Ref		Ref	
No	9.34 (5.71, 13.06)	< 0.001	3.45 (− 0.11, 7.01)	0.074
Hypertension history (%)				
Yes	Ref		Ref	
No	2.87 (0.57, 5.18)	0.021	0.17 (− 1.71, 2.05)	0.861
Sleep disorder				
Yes	− 3.81 (− 6.10, − 1.52)	0.003	− 3.61 (− 5.89, − 1.34)	0.006
No	Ref		Ref	

The parameters adjusted in multivariable linear regression included age, race/ethnicity, gender, married/live with partner status, education level, poverty income ratio, obesity, alcohol use, DM history, hypertension history, and sleep disorder

### The relationship between vitamin D and sleep disorders

Serum vitamin D concentrations were divided into quartile portions according to quartiles: Q1 (< 44.99 nmol/L), Q2 (44.99–59.83 nmol/L), Q3 (59.84–75.99 nmol/L), and Q4 (> 75.99 nmol/L). Table 3 details the odds ratios (ORs) for sleep disorders across these vitamin D concentrations, as per logistic regression analysis. Initially, Model 1, without covariate adjustment, revealed a significant link between lower vitamin D concentrations (Q1, Q2, and Q3) and increased incidence of sleep disorders (p-values: 0.0015, 0.0096, 0.0316, respectively). Model 2, accounting for age, gender, race, and other factors, upheld this association, with significant *p*-values in the first three quartiles (Q1 < 0.001, Q2 0.0017, and Q3 0.012). The comprehensive Model 3 showed that participants in Q1 had a notably higher prevalence of sleep disorders compared to those in Q4 (OR 1.52, 95% CI 1.10–2.10, trend *p* = 0.0139). Figure 2, with multivariable-adjusted spline curves, illustrates this relationship. It was found that lower vitamin D concentrations were consistently negatively associated with sleep disorders prevalence (non-linearity *p* > 0.05).

**Table 3:** Association between serum vitamin D quartiles and sleep disorders

	Model 1	Model 2	Model 3
	OR (95% CI) *p*-value	OR (95% CI) *p*-value	OR (95% CI) *p*-value
Sleep disorders			
Quartile 1	1.58 (1.21, 2.07)**	1.86 (1.39, 2.50)***	1.52 (1.10, 2.10)*
Quartile 2	1.58 (1.13, 2.21)**	1.87 (1.30, 2.70)**	1.72 (1.16, 2.56)*
Quartile 3	1.35 (1.03, 1.77)*	1.45 (1.10, 1.93)*	1.45 (1.05, 2.00)*
Quartile 4	Ref	Ref	Ref
* p* for trend	0.00184	0.00115	0.013879

Model 1, no covariates were adjusted; Model 2, age, sex, and race were adjusted; Model 3, Model 2 plus additional adjustment for the married/live with partner status, education level, poverty income ratios, smoking status, alcohol use, BMI, hypertension, and diabetes were adjusted; a *p* < 0.05 was considered statistically significant

*95% CI* 95% confidence interval, *OR* odds ratio

^*^p < 0.05, **p < 0.01, and ***p < 0.001

### MR of vitamin D and sleep disturbances

**Fig. 3:**
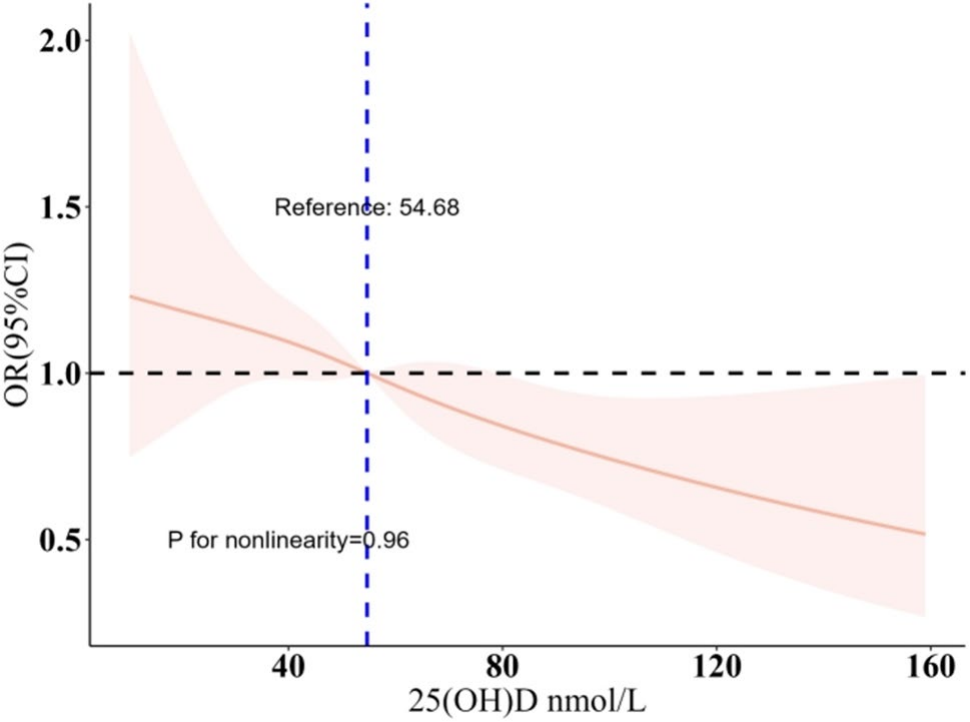
Adjusted multivariable spline curves depicting the relationship between serum vitamin D concentrations and sleep disorders, as evaluated through a spline regression model

**Fig. 4:**
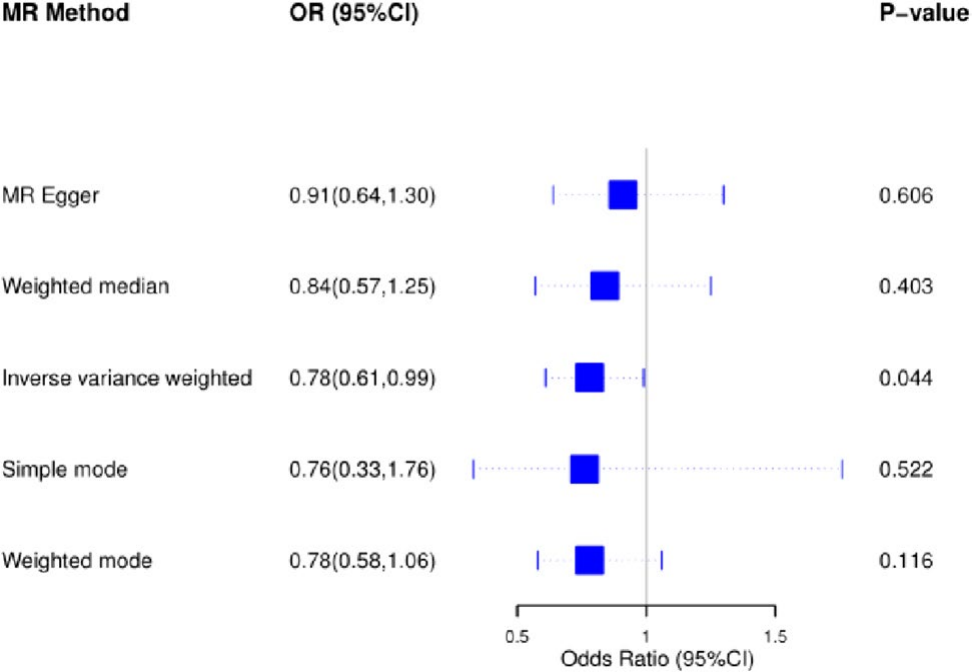
MR results of serum vitamin D and sleep disturbances

**Figure5:**
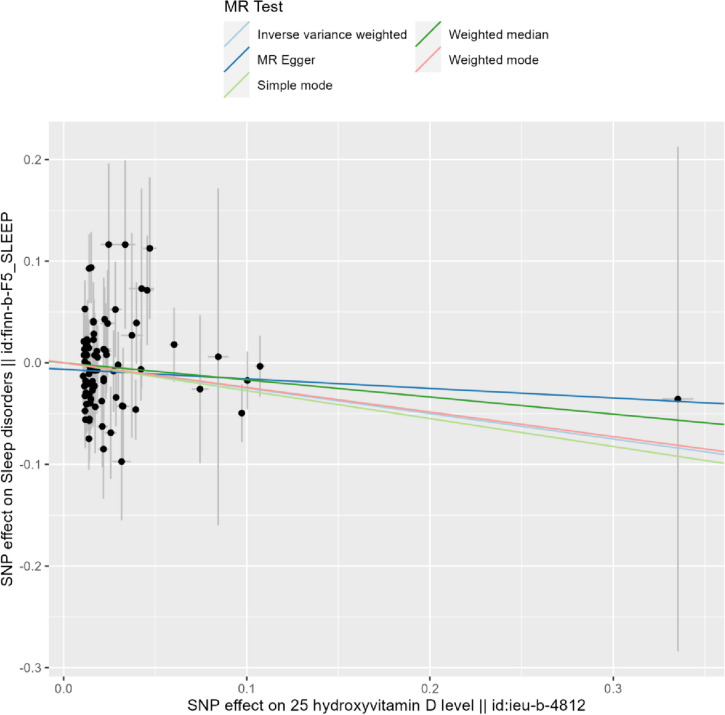
Scatter plot of serum vitamin D and sleep disturbance

The study evaluated the causal effects of genetically predicted vitamin D on sleep disorders using five different Mendelian Randomization (MR) methods. The findings suggested a relationship where higher serum vitamin D concentrations were linked to a reduced risk of sleep disorders (Fig. 3). Specifically, an increase in serum vitamin D concentrations was associated with a 22% decrease in the risk of sleep disorders in high-risk individuals compared to those at low risk. The Inverse Variance Weighted (IVW) method indicated an odds ratio (OR) of 0.78 (95% CI 0.61–0.99, *p* = 0.044) for sleep disturbances, but no significant correlations were found using the other four methods. These results are illustrated in Figs. 4 and 5.

### Tests for pleiotropy and heterogeneity

The MR-Egger regression intercept in the analysis was − 0.006 with a *p*-value of 0.243, indicating no significant horizontal pleiotropy, as detailed in Table 4. Furthermore, heterogeneity tests were conducted, and both the MR-Egger and inverse variance-weighted methods yielded *p*-values greater than 0.05. This suggests the absence of heterogeneity in the data, as presented in Table 5.

**Table 4:** Polygenic analysis of serum vitamin D genetic variations in sleep disorders in a GWAS dataset

GWAS dataset	MR-Egger intercept		
	Egger_intercept	SE	*p*-val
Sleep disorders	− 0.006	0.005	0.243

**Table 5:** Heterogeneity testing of serum vitamin D genetic variations in sleep disorders in a GWAS dataset

Method	MR-Egger intercept		
	*Q*	*Q*_df	*Q*_pval
MR Egger	90.589	87	0.375
Inverse variance weighted	92.025	88	0.364

### Single SNP effect analysis

**Fig. 6:**
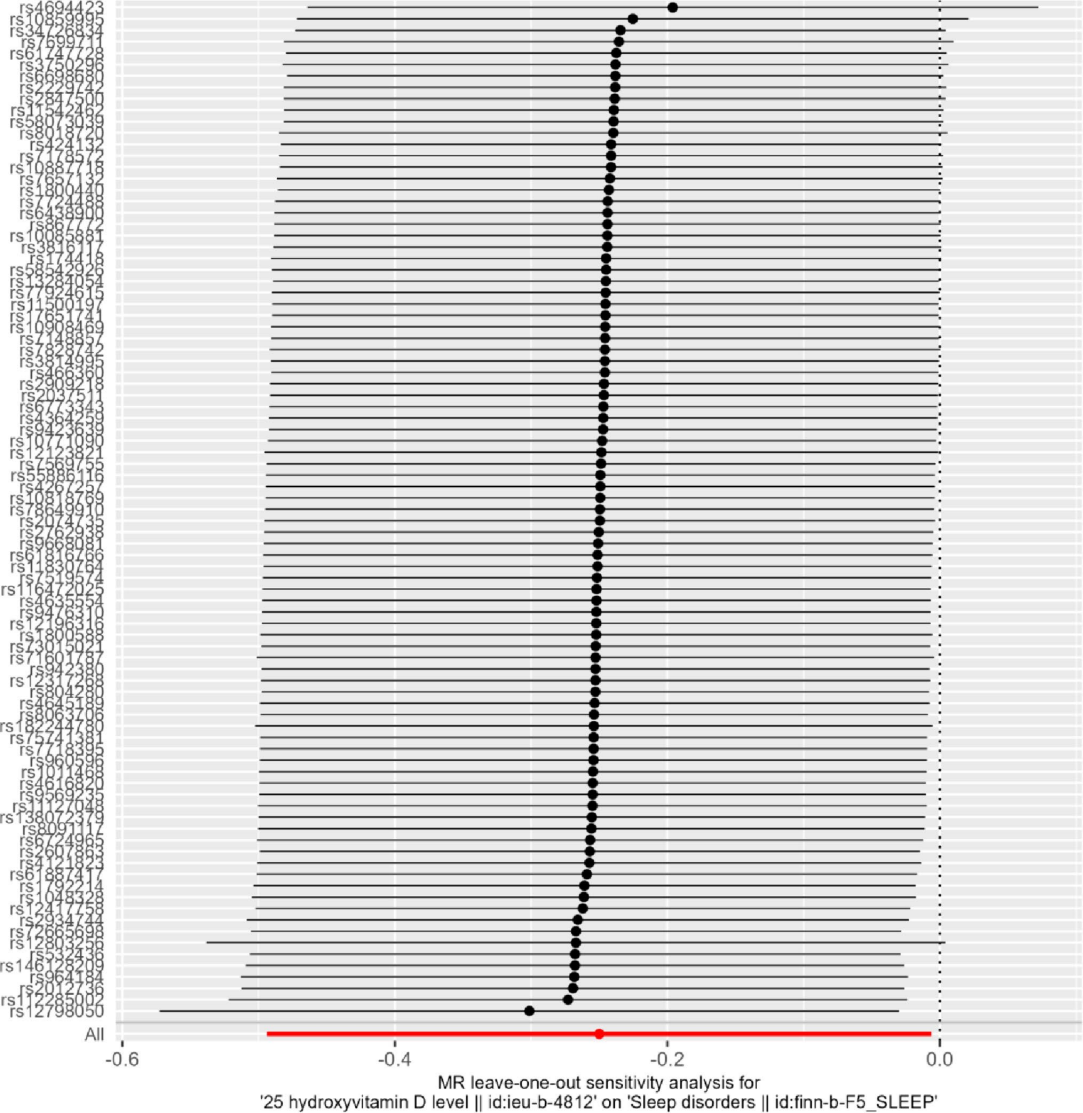
Leave-one-out analysis of the causal association of serum vitamin D and sleep disturbance

**Fig. 7:**
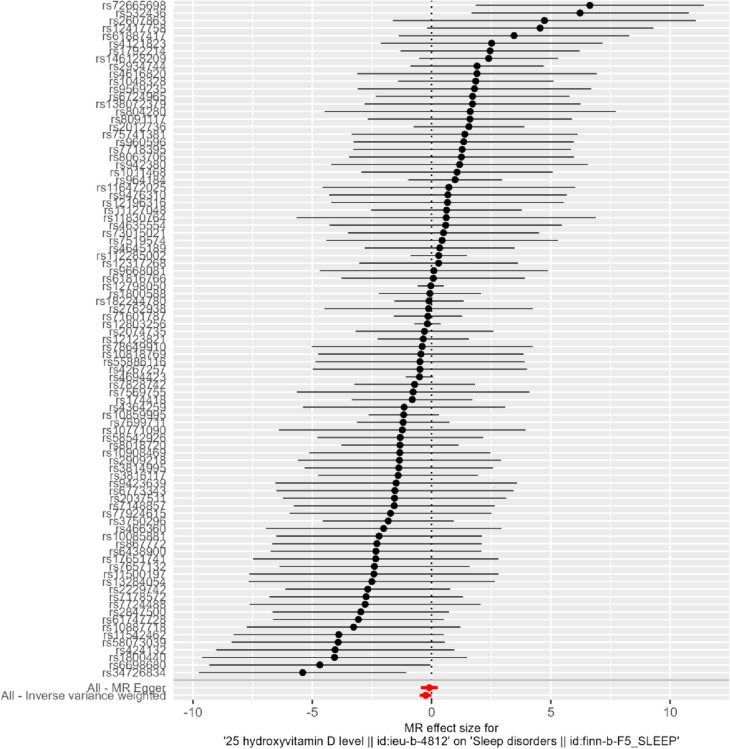
Forest plot of serum vitamin D SNPs associated with sleep disturbances

The forest plot in Fig. 6 and the leave-one-out sensitivity analysis in Fig. 7 of this study provide insights into the relationship between vitamin D-related SNPs and sleep disorders. These analyses strengthen the credibility of the study’s conclusions, indicating that individual vitamin D SNPs do not exhibit significant bias in their effects on sleep disorders. This adds robustness to the overall findings regarding the influence of vitamin D on sleep disturbances.

## Discussion

According to our knowledge, this study is the first of its kind to comprehensively explore the link between serum vitamin D concentrations and the risk of sleep disturbances, utilizing data from large-scale observational studies and genetic data through Mendelian Randomization (MR) analysis. It delves into the relationship and potential causal connection between sleep disturbances and serum vitamin D concentrations. The findings indicate an inverse correlation between serum vitamin D concentrations and sleep disturbances, suggesting a possible negative linear relationship between the two.

This study explores the relationship between serum vitamin D concentrations and sleep disorders, utilizing data from the NHANES 2011–2016, focusing on individuals aged 20–50. After adjusting for various covariates, a negative correlation was found between serum vitamin D concentrations and the occurrence of sleep disorders. Multivariate adjusted spline regression was used to confirm the negative linear relationship. These findings align with previous studies that emphasize the importance of serum vitamin D in sleep quality [24, 25]. The use of the NHANES database, covering a broad population, lends universal and representative validity to the study. Understanding the link between vitamin D concentrations and sleep disorders is significant for clinical value and health guidance. Additionally, the study employs large-scale GWAS data to further investigate the causal relationship, with IVW results supporting this connection. Vitamin D receptors (VDRs) are integral to the diverse biological actions of vitamin D, with their presence identified in the central nervous system, including regions implicated in sleep regulation. The contribution of vitamin D to sleep disorders might be attributed to the widespread distribution of its receptors across various brainstem regions, notably within sections of the hypothalamus, recognized for their critical role in governing sleep regulation. Numerous studies, both domestically and internationally, support the notion that serum vitamin D deficiency leads to sleep disturbances [10, 14]. For instance, a South Korean cross-sectional study found a notable link between vitamin D deficiency and symptoms like daytime sleepiness and reduced sleep duration [26]. Additionally, another study demonstrated that vitamin D supplementation significantly improved sleep duration and decreased sleep disturbances in 20–50 year-olds with sleep disorders [14, 27]. In a research involving 81 American patients with sleep disorders, a higher prevalence of daytime sleepiness was observed among those with vitamin D deficiency [28].

Sleep disorders are a very common problem in today’s society and place a significant burden on individuals, families, and society. Our observational studies showed a negative linear association between serum vitamin D insufficiency and sleep disturbances, and MR analyses in this study also showed a negative correlation between serum vitamin D and sleep disturbances. Although the exact mechanism between sleep disturbance and serum vitamin D concentrations is unknown, the underlying mechanisms are as follows. On the one hand, when sleep disorders are seen as a consequence of vitamin D deficiency, one hypothesis is that prostaglandin D2 (PD 2), as a central regulator of sleep, may lead to obstructive sleep apnea. Vitamin D downregulates cyclooxygenase-2, the rate-controlling enzyme for PD 2 production, in prostate tissue, suggesting that vitamin D insufficiency may lead to an increase in circulating PD 2, leading to sleep disturbance [29, 30]. On the other hand, vitamin D deficiency may lead to musculoskeletal pain, which can lead to poor sleep quality and reduced sleep duration [31, 32]. Sleep quality exhibited a significant enhancement following vitamin D supplementation [27]. Vitamin D receptors (VDRs) play a pivotal role in the multifaceted biological functions of vitamin D, as evidenced by their identification in the central nervous system, encompassing areas involved in sleep regulation. The involvement of vitamin D in sleep disorders may be ascribed to the extensive dissemination of its receptors throughout diverse brainstem regions, particularly within segments of the hypothalamus, which are acknowledged for their essential role in sleep regulation [33–35]. In addition, animal experiments have shown that in the central nervous system, there are several vitamin D-specific binding sites arranged along neurons, symmetrically distributed with sleep-inducing regions [35].

Our study reinforces the significant negative correlation between serum vitamin D concentrations and sleep disturbances, in line with prior research. Additionally, our MR analysis indicates a significant link between serum vitamin D and sleep disturbances. While a direct causal relationship between vitamin D deficiency and sleep disturbances is not fully clarified, the evidence consistently suggests a negative association across various demographics, including children and older adults [10, 24, 25, 36].

This study corroborates a link between lower vitamin D concentrations and a heightened risk of sleep disorders, emphasizing the significance of vitamin D supplementation in the treatment of these conditions for healthcare professionals [37]. Additionally, the findings underscore the need for public health measures to increase awareness about the adverse effects of vitamin D deficiency.

Our study has several limitations. First, the cross-sectional study and MR analysis involved different ethnic groups, possibly leading to population heterogeneity, and future research should be conducted within the same population. Second, the reliance on self-reported sleep disturbance measures may introduce bias. Third, our data do not differentiate between types of sleep disorders, such as obstructive sleep apnea, insomnia, or delayed sleep phase disorder [38]. Due to the unavailability of direct seasonal data in the NHANES database, we were unable to directly consider the influence of seasonal variations on vitamin D levels in our analysis. This limitation may have hindered a comprehensive assessment of the relationship between vitamin D levels and seasonal changes. Additionally, other potential links between serum vitamin D and sleep disturbances were not explored. Our study did not conduct reverse research, i.e., investigating whether sleep disturbances lead to vitamin D deficiency. Although the relationship between sleep disturbances and vitamin D levels has been widely discussed, our study did not provide direct evidence to support this hypothesis. It is noteworthy that our study mainly focused on exploring the association between vitamin D levels and sleep disturbances, without considering the potential impact of sleep disturbances on vitamin D levels. While we controlled for various confounders, residual confounding is possible. Despite these limitations, our study emphasizes the significance of acknowledging the inverse relationship between vitamin D and sleep disorders.

## Conclusions

Collectively, the findings from this study demonstrate a significant negative correlation between serum vitamin D deficiency and sleep disturbances, as evidenced through both cross-sectional and Mendelian Randomization (MR) analyses. These insights suggest that healthcare professionals should be more vigilant regarding sleep disorders in patients, highlighting the necessity for further comprehensive research to confirm these results and to understand the underlying mechanisms of this association.

### Supplementary Information

Below is the link to the electronic supplementary material.

Supplementary file1 (18.5 KB)

## Data Availability

The data used in this study are available on the National Health and Nutrition Examination Survey website: https://www.cdc.gov/nchs/nhanes/index.htm. All the data used in this study had been publicly available.

## Abbreviations

NHANES: National Health and Nutrition Examination Survey
MR: Mendelian Randomization
OR: Odds ratio
CI: Confidence interval
GWAS: Genome-Wide Association Studies
UHPLC-MS/MS: Ultra-performance liquid chromatography-tandem mass spectrometry
LLOD: Lower limit of detection
BMI: Body mass index
NCHS: National Center for Health Statistics
CDC: Centers for Disease Control and Prevention
DBP: Diastolic blood pressure
SBP: Systolic blood pressure
IRB: Institutional Review Board
SNPs: Single-nucleotide polymorphisms
IVs: Instrumental variables
LD: Linkage disequilibrium
IVW: Inverse variance weighted
VDRs: Vitamin D receptors
PD2: Prostaglandin D2
