# Supplementary material for: Serum vitamin D concentrations and sleep disorders: insights from NHANES 2011–2016 and Mendelian Randomization analysis
Source: Sleep Breath. 2024 May 13;28(4):1679–90. doi: 10.1007/s11325-024-03031-2 (PMC11303418; doi:10.1007/s11325-024-03031-2)
Supplement: Supplementary file 1 — Supplementary file1 (18.5 KB) [file 11325_2024_3031_MOESM1_ESM.pdf]

Supplementary Table 1: Comparison Among Different Vitamin D Groups

Variables	Q1 N=1686(25%)	Q2 N=1226(25%)	Q3 N=1085(25%)	Q4 N=916(25%)	P value
Age					0.002
20-29	601 (35%)	405 (33.1%)	337(31%)	265 (29%)	
30-39	548 (33%)	414 (33.7%)	333(30%)	283 (31/%)	
40-50	537 (32%)	407 (33.2%)	415 (38%)	368 (40%)	
Race/ethnicity					<0.001
Mexican American	233 (14%)	241 (20%)	140 (13%)	49 (5.3%)	
Non-Hispanic white	292 (17%)	446 (36%)	551 (51%)	630 (68.8%)	
Non-Hispanic Black	695 (41%)	185 (15%)	110 (10%)	52 (5.7%)	
Other	466 (28%)	354 (29%)	284 (26%)	185 (20.2%)	
Gender					<0.001
Male	821 (49%)	688 (56%)	550 (51%)	405 (44%)	
Female	865 (51%)	538(44%)	535 (49%)	511 (56%)	
Married/live with partner					<0.001
Yes	832 (49%)	741 (60%)	692 (64%)	581 (63%)	
No	854 (51%)	485 (40%)	393 (36%)	335 (37%)	
Education					<0.001
Below high school	311 (18%)	241 (19%)	191 (17.6%)	117 (12.7%)	
High School or above	1375 (82%)	985 (81%)	894 (82.3%)	799 (87.2%)	
Poverty income ratio					<0.001
Poor	626 (37%)	433 (35%)	330 (30%)	267 (29%)	
Not poor	1060 (63%)	793 (65%)	755 (70%)	649 (71%)	
Obesity					<0.001
Yes	748 (44%)	450 (37%)	337 (31%)	223 (24%)	
No	938 (56%)	776 (63%)	748 (69%)	693 (76%)	
Smoking					0.2
Yes	420 (25%)	268 (22%)	246 (22%)	218 (23%)	
No	1266 (75%)	958 (78%)	839 (78%)	698 (77%)	
Alcohol use					<0.001
Yes	1210 (72%)	922 (75%)	871 (80%)	749 (82%)	
No	475 (28%)	303 (25%)	214 (20%)	165 (18%)	
Hypertension history					0.005
Yes	447 (27%)	252 (20%)	218 (20%)	195 (21%)	
No	1239 (73%)	974 (80%)	867 (80%)	721 (79%)	

DM history					<0.001
Yes	174 (10%)	77 (6.0%)	71 (6.5%)	39 (4.3%)	
No	1509 (90%)	1149 (94%)	1014 (93.5%)	876 (95.7%)	
Sleep disorder					0.008
Yes	244 (14%)	174 (14%)	154 (14%)	118 (13%)	
No	1442 (86%)	1052 (86%)	931 (86%)	798 (87%)	
